# Epigenetic Modulation of Human Induced Pluripotent Stem Cell Differentiation to Oligodendrocytes

**DOI:** 10.3390/ijms17040614

**Published:** 2016-04-22

**Authors:** Panagiotis Douvaras, Tomasz Rusielewicz, Kwi Hye Kim, Jeffery D. Haines, Patrizia Casaccia, Valentina Fossati

**Affiliations:** 1 The New York Stem Cell Foundation Research Institute, New York, NY 10032, USA; pdouvaras@nyscf.org (P.D.); trusielewicz@nyscf.org (T.R.); 2 Department of Neuroscience, Icahn School of Medicine at Mount Sinai, New York, NY 10029, USA; kwihyekim@gmail.com (K.H.K.); jefferyhaines@gmail.com (J.D.H.)

**Keywords:** human induced pluripotent stem cells, oligodendrocyte differentiation, histone modifications

## Abstract

Pluripotent stem cells provide an invaluable tool for generating human, disease-relevant cells. Multiple sclerosis is an inflammatory demyelinating disease of the central nervous system, characterized by myelin damage. Oligodendrocytes are the myelinating cells of the central nervous system (CNS); they differentiate from progenitor cells, and their membranes ensheath axons, providing trophic support and allowing fast conduction velocity. The current understanding of oligodendrocyte biology was founded by rodent studies, where the establishment of repressive epigenetic marks on histone proteins, followed by activation of myelin genes, leads to lineage progression. To assess whether this epigenetic regulation is conserved across species, we differentiated human embryonic and induced pluripotent stem cells to oligodendrocytes and asked whether similar histone marks and relative enzymatic activities could be detected. The transcriptional levels of enzymes responsible for methylation and acetylation of histone marks were analyzed during oligodendrocyte differentiation, and the post-translational modifications on histones were detected using immunofluorescence. These studies showed that also in human cells, differentiation along the oligodendrocyte lineage is characterized by the acquisition of multiple repressive histone marks, including deacetylation of lysine residues on histone H3 and trimethylation of residues K9 and K27. These data suggest that the epigenetic modulation of oligodendrocyte identity is highly conserved across species.

## 1. Introduction

Over the past decade, advances in stem cell biology led to the possibility of reprogramming somatic cells to a pluripotent state equivalent to that of embryonic stem cells (ESCs). Induced pluripotent stem cells (iPSCs) have been extensively used since their discovery to generate any desired somatic cell type [1]. iPSC technology is emerging as a particularly useful tool for understanding diseases, such as neurodegenerative disorders, that lack a good animal model. While studies have been hampered by the limited access to primary brain cells, only through highly invasive procedures, the differentiation of iPSCs offers the unprecedented opportunity of obtaining disease-relevant cells in large numbers [2,3,4]. Notably, building on pioneering studies [5,6], our laboratory has recently developed an efficient differentiation protocol to generate iPSC-derived oligodendrocyte progenitor cells (OPCs) and oligodendrocytes [7].

OPCs are the myelinating cells of the central nervous system (CNS). They differentiate into highly arborized oligodendrocytes, expressing myelin-specific proteins and reaching out with their processes to proximal nerve fibers. Recognition and contact with axonal targets induce the synthesis of lipid-rich lamellae of myelin and wrapping of segments of axons into a compact multilayer spiral. This creates a highly specialized insulation that allows for the fast and efficient transmission of electrical signals [8]. The importance of myelin is evident in pathological conditions, such as multiple sclerosis (MS), in which its damage results in slower impulse transmission, more vulnerable axons, functional deficits and eventually irreversible axonal degeneration. Genetic deletion of oligodendrocyte-specific proteins, such as 2′,3′-cyclic nucleotide phosphohydrolase (CNP) in transgenic mice, does not lead to any apparent defect in myelin, but causes profound axonal degeneration in the long-term, manifested through progressive accumulation of neurological symptoms (e.g., convulsions, gait abnormalities, weakness) and ultimately precocious death [9]. These findings suggest that oligodendrocytes and myelin are essential for neuronal health and optimal CNS functioning, and recent evidence highlighted the importance of myelin in cognition, memory and learning of complex skills [10]. Thus, a thorough understanding of the processes leading to the generation of myelinating oligodendrocytes is of high relevance to a vast number of neurological and psychiatric disorders. Most of our knowledge on the processes of oligodendrocyte differentiation and maturation has been extensively investigated, and their major regulators have been identified in rodent models [11,12,13,14,15]. It is now clear that the oligodendrocyte lineage commitment results from subsequent waves of regulation of gene expression that first reduce precursor markers and inhibitory signals and then increase the expression of mature markers. This is achieved by the deposition of specific repressive and activating marks on lysine residues in the tail of histone H3 [11,15,16]. In particular, deacetylation of lysine residues on H3 has been involved in the early repression of transcriptional inhibitors of myelin genes at the progenitor state, as cells stop proliferating and initiate a transcriptional program of differentiation [15,17,18]. The repressive trimethylation of K27 (H3K27me3) by the enzyme EZH2 has been shown by our group and others to critically regulate the repression of neuronal genes during the transition from neural stem cells (NSCs) to OPCs [19], while the deposition of the trimethyl mark on H3K9 (H3K9me3) has been shown to repress the expression of genes modulating the electrical properties of oligodendrocyte progenitors, which need to be silenced during the maturation into electrically-silent myelinating oligodendrocytes [11]. While these studies identified the importance of several epigenetic processes for developmental myelination in rodents, the translation to human cells is fundamental, and this is the goal of this study.

In this study, we asked whether the molecular mechanisms of oligodendrocyte differentiation are conserved among species, with an emphasis on the investigation of the repressive changes in histones occurring during oligodendrocyte lineage commitment and differentiation of human iPSCs.

## 2. Results

### 2.1. Histone Deacetylases, Histone Acetyltransferases and Histone Methyltransferases in Human Oligodendrocyte Differentiation from Embryonic Stem Cells

**Figure 1:**
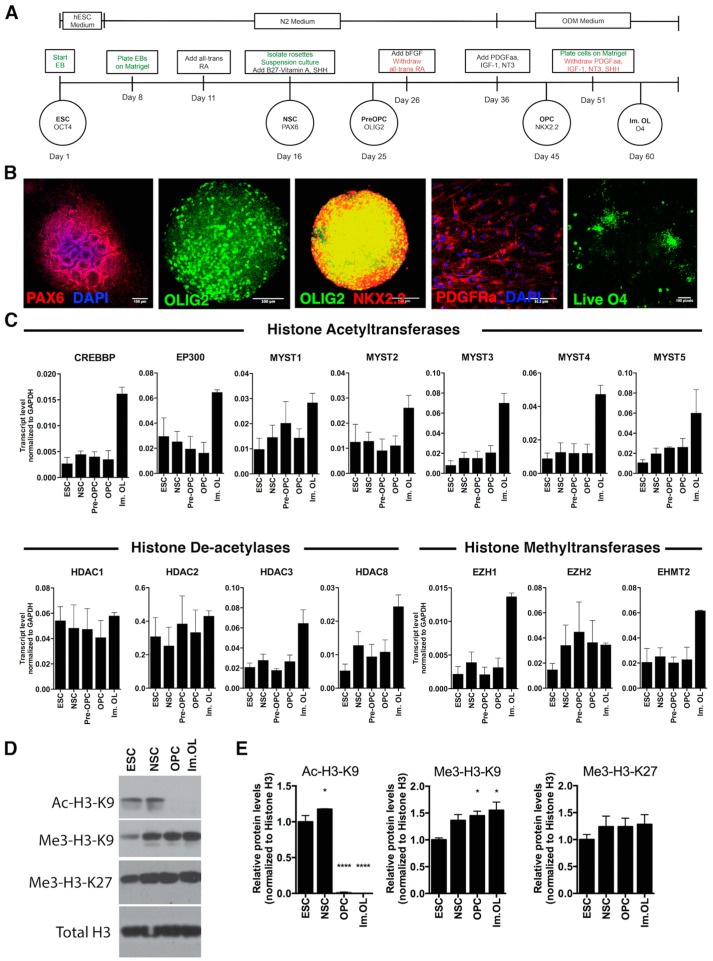
Expression pattern of histone modifiers and relative histone marks during oligodendrocyte differentiation of ESCs. (**A**) Diagram depicting the major steps of oligodendrocyte differentiation with the corresponding cellular markers that peak at different days of differentiation. In green are described the technical procedures and in red the reagent withdrawal. ESC: embryonic stem cell; EB: embryoid body; NSC: neural stem cell; OPC: oligodendrocyte progenitor cell; Im. OL: immature oligodendrocyte; (**B**) Representative images showing immunofluorescent detection of oligodendrocyte-lineage markers during differentiation. From **left** to **right**: PAX6 in rosette-like structures, OLIG2 in free-floating spheroids, coexpression of OLIG2 and NKX2.2 in free-floating spheroids, PDGFRα in single cells after trituration of spheroids, live O4 staining in single cells; scale bars: 100 µm; (**C**) Transcriptional levels of histone acetyltransferases, deacetylases and methyltransferases during oligodendrocyte differentiation. The bars are the mean ± SEM relative to GAPDH; (**D**) Western blot analysis of specific epigenetic marks on H3 at different days of differentiation; (**E**) Quantification of Western blots was performed by determining the relative expression level of (from left to right) Ac-H3-K9, Me3-H9-K9 and Me3-H9-K27 relative to histone H3. Data were normalized to ESC, and results are expressed as the mean ± SEM (* *p* < 0.05; **** *p* < 0.0001).

Human ESCs’ (hESCs) differentiation into oligodendrocytes is a well-defined stepwise process that recapitulates embryonic development (Figure 1A). Under specific culture conditions, hESCs were induced to neural differentiation and by Day 16 re-arranged into rosette structures, expressing PAX6, a typical marker of NSCs. NSCs, in the presence of sonic hedgehog (SHH) and all-trans retinoic acid (RA), upregulated OLIG2, a marker of the early oligodendrocyte lineage (pre-OPC). The addition of platelet-derived growth factor (PDGF-AA), Insulin-like growth factor-1 (IGF-1), triiodothyronine (T3) and neurotrophin-3 (NT-3) from Day 36 resulted in the expression of NKX2.2 on OLIG2 progenitors, a stage described as early oligodendrocyte progenitor cells (OPCs), which eventually express platelet-derived growth factor receptor alpha (PDGFRα). Removal of the growth factors led to further differentiation into immature oligodendrocytes (Im. OL), characterized by the expression of the sulfated glycolipid antigen identified by the O4 antibody. The major steps of oligodendrocyte differentiation were assessed by immunofluorescent analysis, which highlighted the characteristic morphology of cells at each stage of maturation, including bipolar OPCs and ramified Im. OL (Figure 1B).

Others and we have previously shown that the acetylation state of lysine residues on histone H3 is high in proliferating oligodendrocyte progenitor cells and is catalyzed by histone acetyltransferases (HATs, which place the acetyl group on lysines), while the early stages of differentiation are characterized by the removal of these activating marks catalyzed by histone deacetylases (HDACs) [20,21]. Lineage progression is further characterized by repressive histone methylation of lysine residues K9 and K27, which is catalyzed by specific histone methyltransferases for K9 (e.g., EHMT2) [22] and K27 (e.g., EZH2) [23]. As a first step towards the characterization of epigenetic changes during oligodendrocyte differentiation of human stem cells, we assessed the transcript levels of histone acetyltransferases, histone deacetylases and histone methyltransferases in the sequential stages described above (Figure 1C).

Consistent with the previous report of increased acetylation at myelin gene promoters and enhancers during differentiation [24], expression of the acetyltransferase genes *CREBBP*, *EP300* and MYST family showed an increase at the final Im. OL stage of differentiation. On the other hand, the specific activity of class I HDACs (HDAC-1, -2, -3, -8) has been implicated in the development of myelinating oligodendrocytes to initiate chromatin compaction [15]. Transcript levels of *HDAC3* and *HDAC8* progressively increased from NSCs to Im. OL, while *HDAC1* and *HDAC2* expression remain similar at the various stages of the differentiation.

Next, we examined the expression levels of the major enzymes responsible for the methylation of H3K9 and H3K27. Our results were consistent with previous reports [19] on increased levels of the H3K27-specific methyltransferase *EZH2* during the transition from the NSC stage to the OLIG2 early progenitors’ stage. In addition, we identified a marked increase of the H3K9-specific methyltransferase *EHMT2* (also known as *G9a*), as progenitors mature into Im. OLs.

To determine whether these patterns of gene expression were associated with actual changes in histone marks, we also performed a Western blot analysis of protein extracts from NSCs, OPCs and Im. OLs (Figure 1D). We have previously shown that rodent oligodendrocyte progenitors in culture and in developing white matter tracts are characterized by histone acetylation [12,15] and that their differentiation into oligodendrocytes requires HDAC activity to remove the acetyl groups from nucleosomal histone tails [15,17]. Oligodendrocyte differentiation is accompanied by additional repressive modifications on histone H3, including: trimethylation of H3K27, which is prominent already at the OPC stage and di- and tri-methylation of H3K9 residues [11], functionally responsible for the repression of alternative lineage traits and electrical excitability during the transition of OPC to myelinating oligodendrocytes [11]. In line with the rodent data, in human cells, we detected increased levels of H3K27me3 from NSCs to immature OL and significantly decreased levels of histone acetylation (*i.e.*, H3K9ac) at the early stages of OPC differentiation, associated with increasing levels of H3K9me3 during OPC maturation (Figure 1E).

### 2.2. The Epigenetic Signature Is Conserved in Induced Pluripotent Stem Cell-Derived Oligodendrocytes

**Figure 2:**
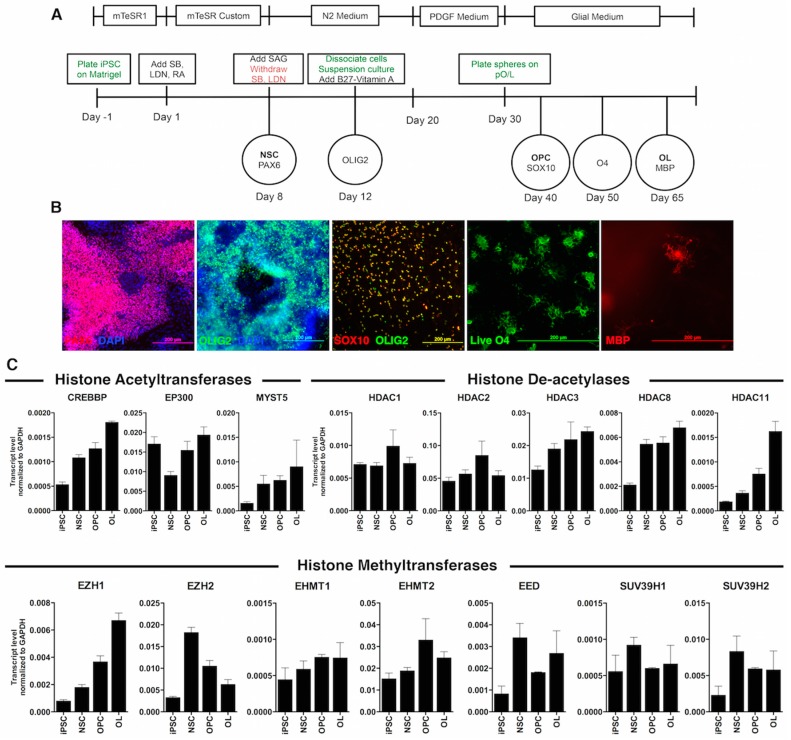
Transcriptional profile of histone modifiers during differentiation of human induced pluripotent stem cells (iPSCs) into oligodendrocytes. (**A**) Schematic representation of the oligodendrocyte differentiation protocol, including oligodendrocyte lineage markers along the differentiation. In green are described the technical procedures and in red the reagent withdrawal; (**B**) Representative images depicting the most important oligodendrocyte lineage markers after immunofluorescent labeling. From **left** to **right**: PAX6, OLIG2 in the 3D structures, SOX10 and OLIG2 staining in cells growing out of the plated aggregates, live O4 staining of cells migrating out of the spheres, myelin basic protein (MBP) staining of oligodendrocytes at the end of the differentiation; scale bars: 200 µm; (**C**) Quantitative RT-PCR showed differences in the transcript levels of enzymes responsible for acetylation, deacetylation and methylation during oligodendrocyte lineage commitment. The bars are the mean ± SEM, relative to GAPDH levels.

Next, we differentiated a human iPSC line through the oligodendrocyte protocol that we recently published [7,25]. This protocol (Figure 2A) was based on the dual SMAD inhibition for neural induction and achieved an accelerated differentiation through the critical stages of NSCs, pre-OPCs, OPCs and Im. OL. Moreover, it allowed the final differentiation of Im. OL to mature oligodendrocytes expressing myelin basic protein (MBP). Immunofluorescent analysis confirmed the expression of the typical markers, as the cells progress from NSCs at Day 8 to oligodendrocytes at Day 65 (Figure 2B).

The analysis of HATs, HDACs and K9 and K27 histone methyltransferases was overall consistent with the findings obtained from ESC-derived cells. We observed a steady increase of transcript levels for the HATS: *CREBBP* and *MYST5*. We also confirmed that *HDAC8* was upregulated as early as the NSC stage, while *HDAC1* and *HDAC2* did not display significant patterns of expression across the lineage. In agreement with published evidence on the critical importance of HDAC11 activity for oligodendrocyte development in rats [26], we detected increased levels of *HDAC11* only in MBP^+^ mature oligodendrocytes.

The levels of the EED and EZH2, subunits of the enzymatic complex responsible for H3K27 methylation, peaked at the NSC stage and slowly tapered off as OPC differentiated. Surprisingly, EZH1 expression was increased in both ESC-derived Im. OL and iPSC-derived OL (Figure 1C and Figure 2C).

Among the enzymes responsible for the di- and tri-methylation of H3K9, EHMT2 expression increased at the OPC and mature oligodendrocyte stages; SUV39H1 expression remained constant over time; and SUV39H2 expression slightly increased from the NSC stage (Figure 2C).

**Figure 3:**
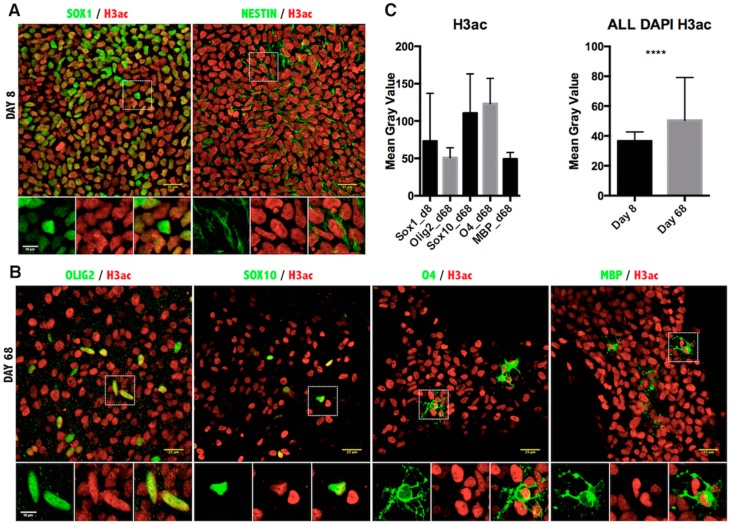
Immunofluorescence analysis of histone H3 pan-acetylation in iPSC-derived oligodendrocyte lineage cells. (**A**) Representative images of Day 8 of the oligodendrocyte differentiation protocol co-stained for NSC markers SOX1, NESTIN and the pan-acetylated histone 3 antibody; (**B**) Images from Day 68 of differentiation co-stained for oligodendrocyte markers OLIG2, SOX10, O4, MBP and H3ac antibody. Scale bar = 25 μm. The magnified view of the broken line box area appears as the inset with the individual channels and the merged image. Scale bar = 10 μm; (**C**) Quantitation of the immunofluorescence signal as the mean gray value in cells expressing the indicated markers (**left**) or in all DAPI-positive cells per field (**right**). Results are represented as the mean with the SD. **** *p* < 0.0001.

**Figure 4:**
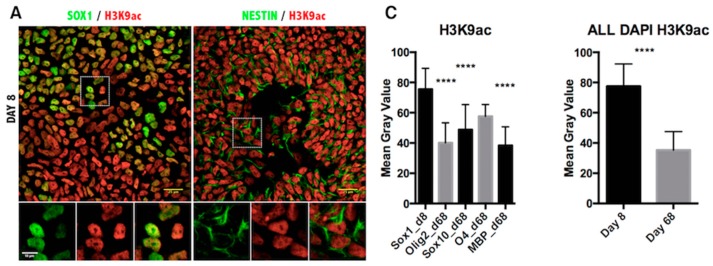

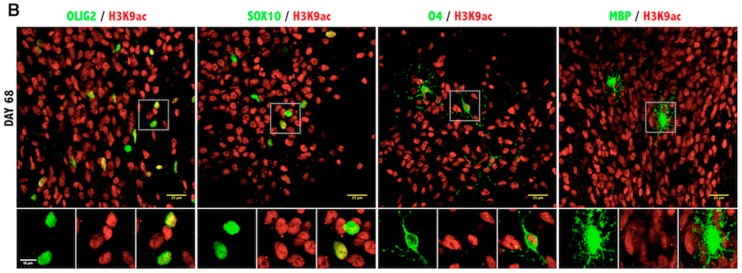
Immunofluorescence analysis of histone H3 lysine 9 acetylation in iPSC-derived oligodendrocyte lineage cells. (**A**) Representative images of Day 8 of the oligodendrocyte differentiation protocol co-stained for NSC markers SOX1, NESTIN and the H3K9ac antibody; (**B**) Images from Day 68 of differentiation co-stained for oligodendrocyte markers OLIG2, SOX10, O4, MBP and H3K9ac antibody. Scale bar = 25 μm. The magnified view of the broken line box area appears as the inset with the individual channels and the merged image. Scale bar = 10 μm; (**C**) Quantitation of the immunofluorescence signal as the mean gray value in cells expressing the indicated markers (**left**) or in all DAPI-positive cells per field (**right**). Results are represented as the mean with the SD. **** *p* < 0.0001.

**Figure 5:**
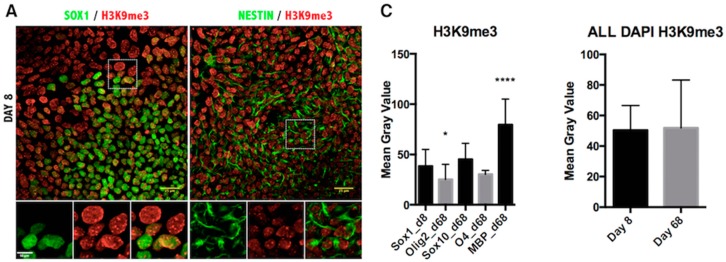

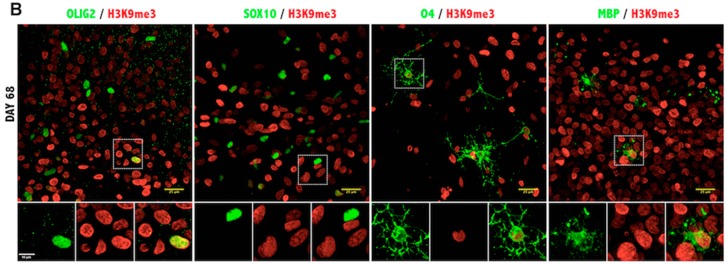
Immunofluorescence analysis of histone H3 lysine 9 trimethylation in iPSC-derived oligodendrocyte lineage cells. (**A**) Representative images of Day 8 of the oligodendrocyte differentiation protocol co-stained for NSC markers SOX1, NESTIN and the H3K9me3 antibody; (**B**) Images from Day 68 of differentiation co-stained for oligodendrocyte markers OLIG2, SOX10, O4, MBP and H3K9me3 antibody. Scale bar = 25 μm. The magnified view of the broken line box area appears as the inset with the individual channels and the merged image. Scale bar = 10 μm; (**C**) Quantitation of the immunofluorescence signal as the mean gray value in cells expressing the indicated markers (**left**) or in all DAPI-positive cells per field (**right**). Results are represented as the mean with the SD. * *p* < 0.05; **** *p* < 0.0001.

**Figure 6:**
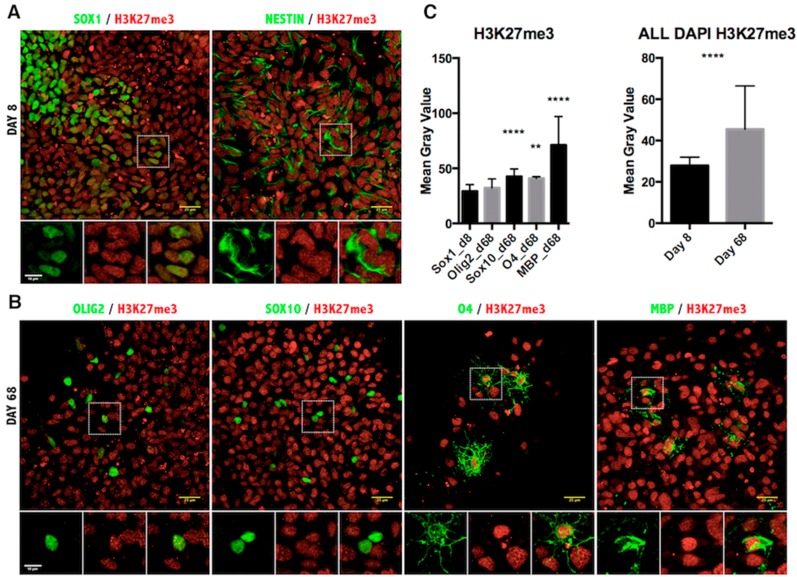
Immunofluorescence analysis of histone H3 lysine 27 trimethylation in iPSC-derived oligodendrocyte lineage cells. (**A**) Representative images of Day 8 of the oligodendrocyte differentiation protocol co-stained for NSC markers SOX1, NESTIN and the H3K27me3 antibody; (**B**) Images from Day 68 of differentiation co-stained for oligodendrocyte markers OLIG2, SOX10, O4, MBP and H3K27me3 antibody. Scale bar = 25 μm. The magnified view of the broken line box area appears as the inset with the individual channels and the merged image. Scale bar = 10 μm; (**C**) Quantitation of the immunofluorescence signal as the mean gray value in cells expressing the indicated markers (**left**) or in all DAPI-positive cells per field (**right**). Results are represented as the mean with the SD. ** *p* < 0.001; **** *p* < 0.0001.

To validate the functional significance of the transcriptional data on histone modifiers, we asked whether the histone marks in differentiated iPSCs would be consistent with the predicted changes of enzymatic activities. For this reason, we performed double immunofluorescence using antibodies specific for each stage-appropriate oligodendrocyte marker and for the post-translational modifications of lysine residues on histone H3 (Figure 3, Figure 4, Figure 5 and Figure 6).

We have previously mentioned that acetylation of lysine residues serves as an activator mark on histone and that it is highly dynamic during oligodendrocyte differentiation, with K9 acetylation being decreased at the promoter of transcriptional inhibitors of oligodendrocyte differentiation [16,17] and possibly acetylation of other K residues being increased at myelin genes [24]. Consistent with the increased HAT levels in OPC progenitors and the limited increased acetylation of myelin genes in OL, confocal imaging and quantification of H3 acetylation (H3ac) intensity revealed an overall increase of H3ac as oligodendrocyte progenitors were generated from NSC, followed by a sharp decrease during differentiation of O4^+^ cells into MBP^+^ OL (Figure 3). Consistent with the notion that K9 residues are critically important for the establishment of repressive methylation and that acetylation of this residue precludes the establishment of repressive marks, we also detected a clear reduction of acetylation of H3K9 (H3K9ac) (Figure 4). Thus, the dynamic changes of acetyl marks on histones were consistent with the expression pattern of the relative enzymatic activities.

Lastly, we assessed the repressive epigenetic marks H3K9me3 (Figure 5) and H3K27me3 (Figure 6). The pattern of H3K9me3 nuclear distribution at Day 8 of differentiation was evident as punctuated fluorescence, indicating the focal formation of heterochromatin at the initial stages of development. As cells differentiated further, the appearance of H3K9me3 changed drastically, staining uniformly the whole nucleus in the MBP^+^ stage of differentiation (Figure 5B) and indicating the massive increase of heterochromatin formation characteristic of mature oligodendrocytes [27]. Quantification of H3K9me3 intensity in the oligodendrocyte nuclei confirmed a significant increase in maturing oligodendrocytes (Figure 5C). Furthermore, quantification of intensity showed a significant increase in the H3K27me3 repressive mark, as well (Figure 6).

## 3. Discussion

Epigenetic repression seems to be a common mechanism to drive the differentiation of different cell-types and is highly conserved from fish to mammals [28]. However, very little is known about the epigenetic identity of human oligodendrocytes, since primary cells have been hardly available for research. The accessibility to human pluripotent stem cells (encompassing both ESCs and iPSCs) provided us with a tool to address the question on the conservation of the epigenetic landscape in humans. Most of the previous studies on the epigenetic changes occurring during the differentiation of OPC into oligodendrocytes had been performed in rodent cells and established that post-translational modifications of histones, such as dynamic modulation of histone acetylation and repressive methylation, regulated the acquisition of the oligodendrocyte identity. Others and we, for instance, reported deacetylation of lysine residues in the tail of histone H3 as critical for the onset of myelination in rats as pharmacological inhibitors of HDACs [12,15] or genetic ablation of HDACs [21] impaired OPCs differentiation. It was suggested that removal of acetyl groups from lysine residues of nucleosomal histones at promoters of genes encoding for transcriptional inhibitors of myelin gene expression would favor oligodendrocyte differentiation [20]. However, it was also clear that at late stages of differentiation (after the inhibitors have been repressed), acetylation of histone H3 at promoters and enhancers of myelin genes facilitates gene expression [24]. We therefore suggested that overall deacetylation is needed during the early stages of OPC differentiation to “remove the differentiation breaks”, and this occurs mostly by deacetylating a critical lysine residue, H3K9. However, acetylation is a relatively unstable mark, driven by the equilibrium between HATs and HDACs, and we reasoned that deacetylation of K9 would be necessary to guarantee access to histone methyltransferases, which can methylate K9 and thereby provide a more stable form of repression, by initiating the formation of heterochromatin [11,29]. The detection of high transcript levels of EZH2, the enzymatic activity responsible for methylation of H3K27, in human NSCs derived either from ESC or iPSC differentiation, was consistent with previous reports on the function of H3K27 trimethylation as critical for the restriction of multipotentiality in ESCs [30] and on the role of EZH2 during the oligodendrocyte lineage choice of NSCs, associated with the repression of astrocyte and neuronal lineage gene [19]. The levels of H3K9 dimethyltransferases (*i.e.*, EHMT2), in contrast, showed a tendency towards increase at later stages of oligodendrocyte differentiation (Figure 1C and Figure 2C). This is consistent with the detection of repressive H3K9 methylation marks in human cells (Figure 5) and with the previous reports in rodent cells [11]. While in human cells, we have not conducted silencing experiments in any of the enzymes, the combined evaluation of knockdown experiments for the distinct methyltransferases in rodent cells, together with the genomic distribution of H3K9me3 and H3K27me3 ChipSeq data suggest unique and redundant roles for K9 HMTs and K27 HMTs [11]. Taken together, our data in rodents suggest that methylation at K9 and K27 residues have very distinct functions and that the H3K27me3 mark, deposited by EZH2, occurs early during the commitment of NSC to the oligodendroglial fate, during the transition from NSCs to OPCs, while the repressive H3K9me3 marks occur later in the oligodendrocyte lineage and serve the function to further silence neuronal genes and channels contributing the electrical properties of OPCs, which need to be silenced during the maturation to oligodendrocytes [11]. This model further implies that oligodendrogenesis results from the repression of neurogenesis and astrogliogenesis and is a unidirectional process, as mature oligodendrocytes cannot revert to an early undifferentiated state. Using two distinct human culture systems, ESC and iPSC, we identified a pattern of epigenetic changes that was consistent between the two human cell systems and in agreement with the rodent results. Histone modifications during differentiation along the OL lineage were reproducible, not altered by the reprogramming process on iPSCs and occurred independently of the culture protocol used for differentiation.

It is widely accepted that histone-specific marks, even at specific residues of a single histone protein, can be associated with multiple enzymes. Although there may be variability in the levels of histone modification enzymes, the end result is the deposition or removal of the specific repressive or activating mark on the histone residues. The fluctuation of transcript levels of enzymes and their kinetics in our qPCR data can probably be explained by the redundancy and/or cell specificity of these enzymes. Furthermore, this apparent discrepancy in the upregulation of both HATs and HDACs (Figure 1C and Figure 2C) could also reflect the existence of alternative mechanisms of activation. It should be noted that the qRT-PCR technique is performed with mRNA isolated from entire cultures and not from purified cell-types, therefore containing contaminant cells, such as neurons and astrocytes. Indeed, in our panel of immunofluorescent images, only a fraction of the total population expressed the OLIG2 marker. Therefore, the definite answer for the epigenetic status of the chromatin should come from the *in situ* identification of the end product, that is the H3ac on the oligodendrocyte lineage cells. The immunofluorescent staining revealed that acetylation of H3K9 was significantly reduced during oligodendrocyte differentiation of iPSCs (Figure 4C), and a similar conclusion could be drawn from the Western blot analysis during ESC differentiation (Figure 1D,E). The removal of the acetyl group from this lysine residue is critical for the deposition of the repressive methylation mark, and in agreement with this determination, H3K9me3 was found to be specifically increased in the MBP-expressing oligodendrocytes (Figure 5C). The other repressive mark, H3K27me3, was significantly increased in the MBP^+^ oligodendrocytes (Figure 6C, left panel), as well as in the total number of cells (DAPI^+^) at Day 68 of differentiation (Figure 6C, right panel). However, H3K27me3 was not significantly increased when only the OLIG2 population was scored; this could have been related to the heterogeneous composition of the OLIG2^+^ population, which includes OPCs, Im. OL and mature oligodendrocytes, as this marker is present at all developmental stages of oligodendrogenesis. Because H3K9me3 is associated with repressive heterochromatin formation, it is important that its nuclear localization changes over the course of differentiation, starting with punctate staining patterns (Figure 5A), presumably representing small heterochromatic foci, and then spreading throughout the nucleus as heterochromatin occupies the majority of the differentiated OL nuclei (Figure 5B) [27].

An additional layer of regulation of gene expression is provided by the microRNAs, which have been shown to be important and critical for oligodendrocyte biology [31]. We suggest that while histone modifications are likely to shape the architecture of the epigenomic landscape associated with lineage-specific choices, the microRNAs serve to fine-tune the levels of specific transcriptional modulators.

Our results clearly underline the importance of epigenetic regulation in the commitment of human OPCs to myelinating oligodendrocytes and suggest that enzymes regulating epigenetic marks should be further investigated as potential targets for treating disorders involving oligodendrocytes and myelin. Of note, we have previously found changes in histone acetylation in brain specimens from patients affected by multiple sclerosis (MS), a chronic demyelinating disease of the CNS [32]. We have also generated several MS-specific iPSC lines, and the comparison of the epigenetic profiles may reveal some novel mechanisms of pathogenesis. Remyelinating therapies represent a current unmet need in the MS field, and many efforts are focusing on stimulating the endogenous OPCs that are still present in the brain of the patients, but fail to differentiate. Targeting the epigenetic landscape, for example, by combining molecules interfering with histone acetylation [33] and increasing H3K9me3 could potentially hold the key to successful treatment.

## 4. Materials and Methods

### 4.1. Human Pluripotent Stem Cell Culture Conditions

One ESC line and one iPSC line were used for this study. WA09 (H9) is an NIH-approved ESC, obtained from the WiCell Research Institute under a material transfer agreement with the Casaccia laboratory. hESCs were grown on gamma-irradiated mouse embryonic fibroblasts (γ-MEF) prepared by Icahn School of Medicine Stem Cell Core Facility in ES medium (DMEM-F12, 20% knock out serum replacement, 1× non-essential amino acids, ß-mercaptoethanol (all from ThermoFisher, Cambridge, MA, USA) supplemented with 10 ng/mL of bFGF (R & D Systems, Minneapolis, MN, USA).

The iPSC line iPSC49026 was generated at the New York Stem Cell Foundation Research Institute by mRNA/miRNA reprogramming technology from skin fibroblasts of a de-identified male healthy donor [34]. Skin biopsy was obtained upon institutional review board approval and receipt of informed consent. iPSCs were maintained in feeder free conditions, seeded onto matrigel-coated plates (BD Biosciences, San Jose, CA, USA) in the presence of mTeSR1 medium (StemCell Technologies, Vancouver, BC, Canada).

### 4.2. Oligodendrocyte Differentiation Protocol from hESCs

Oligodendrocyte differentiation of ESCs was performed following previously-published methods [35] with minor changes. Specifically, embryoid bodies (EBs) were formed by incubating hESC clusters in ES media without bFGF supplement in suspension. At Day 5, EBs were collected and transferred to N2 media (DMEM/F12, 1× N2 supplement (ThermoFisher), 1× non-essential amino acids and 2 µg/mL heparin, Sigma-Aldrich, St. Louis, MO, USA). From Day 8, EBs were plated and maintained on Matrigel-coated plates in N2 media. At Day 11, the media was supplemented with 1 μM all-trans retinoic acid (RA, Sigma-Aldrich) until the detection of neural rosettes around Day 16. Neural rosettes were isolated and maintained in suspension in N2 media supplemented with RA, 1× B-27 without vitamin A (ThermoFisher) and 1 µM purmorphamine (Stemgent, Cambridge, MA, USA) or 100 ng/mL sonic hedgehog (SHH, R & D Systems). On Day 26 of differentiation, RA was removed, and 10 ng/mL bFGF were added in the media for 10 days. From Day 36, the cells were further differentiated in oligodendrocyte differentiation media (ODM), containing N2 medium, 1× B27 without vitamin A and 60 ng/mL triiodo-l-thyroxine (T3, Sigma-Aldrich), adding 10 ng/mL of PDGF-AA (Peprotech, Rocky Hill, NJ, USA), IGF-1 (Peprotech) and NT-3 (Peprotech) and 1 µM purmorphamine or 100 ng/mL sonic hedgehog (SHH). Finally, cells were dissociated by trituration after treatment with accutase (ThermoFisher) and plated onto Matrigel-coated dishes in ODM medium.

### 4.3. Oligodendrocyte Differentiation Protocol from Human iPSCs

Oligodendrocyte differentiation from iPSCs followed the “fast” protocol, as described in our previous paper, where catalogue numbers for all reagents used can be found [7]. Briefly, small iPSC colonies were induced in mTeSR custom medium (mTeSR1 minus 5 factors that sustain pluripotency, Stem Cell Technologies) containing 10 µM SB431542 (Stemgent), 250 nM LDN189193 (Stemgent) and 100 nM all-trans RA (Sigma-Aldrich). From Day 8, cells were cultured in N2 medium (DMEM/F12, NEAA, GlutaMAX 2-mercaptoethanol, penicillin-streptomycin, plus N2 supplement, all from Life Technologies, Carlsbad, CA, USA) adding 100 nM RA and 1 µM smoothened agonist (SAG) (EMD Millipore, Billerica, MA, USA), and at Day 12, they were lifted by accutase (Life Technologies) treatment. Floating spheres were then cultured in N2B27 medium (N2 medium plus B27 without vitamin A and 25 µg/mL insulin) containing RA and SAG until Day 20, when PDGF medium was used to drive oligodendrocyte commitment. PDGF medium: N2B27 medium, PDGF 10 ng/mL, IGF 10 ng/mL, HGF 5 ng/mL (all from R & D Systems), NT-3 10 ng/mL (Millipore), biotin 100 ng/mL, cAMP 1 µM, T3 60 ng/mL (all from Sigma). At Day 30, spheres were plated onto poly-l-ornithine/laminin-coated dishes (poly-l-ornithine, Sigma; natural mouse laminin, Life Technologies) in glial medium, consisting of N2B27 medium plus ascorbic acid 20 µg/mL, HEPES 10 mM (both from Sigma), biotin 100 ng/mL, cAMP 1 µM and T3 60 ng/mL.

### 4.4. RNA Isolation and Quantitative RT-PCR

hESC-derived cells at specific stages of oligodendrocyte differentiation were collected in TRIzol, and RNA was isolated following the manufacturer’s recommendation. RNA was further cleaned using RNEasy Mini Kit (Qiagen, Hilden, Germany). mRNA was reverse-transcribed using qScript (Quanta, Houston, TX, USA), and changes in the transcript level were measured by q-RT-PCR using SYBR-green detector (Quanta).

hiPSC-derived cells at specific stages of oligodendrocyte differentiation were collected in lysis buffer, and RNA was isolated using the RNeasy Plus Mini Kit (Qiagen) with QIAshredder (Qiagen), according to the manufacturer instructions. RNA was quantified using Nanodrop 8000 (Thermo Scientific), and its quality was assessed by the OD_260_/OD_280_ ratio. Zero-point-five micrograms of mRNA were reverse-transcribed using the GoScript Reverse Transcription System (Promega, Madison, WI, USA) using random primers. cDNAs were loaded with SYBR-select master-mix (Life Technologies), and qRT-PCR was performed on Stratagene Mx300P qPCR System (Agilent Technologies, Santa Clara, CA, USA). The melting curves were measured to ensure the specificity of the products. Data were normalized to the housekeeping gene GAPDH and analyzed using the comparative C_T_ method (∆Ct). Values are the means of technical replicates with SEM as the error bars. The primers used are listed in Table 1.

### 4.5. Immunofluorescent Analysis

At specific days during oligodendrocyte differentiation, cells grown on Thermanox plastic coverslips were fixed with 4% paraformaldehyde (PFA) (Santa Cruz Biotechnology, Dallas, TX, USA) for 7 min at room temperature (RT). For immunostaining, cells were washed 3× in PBS-T (PBS with 0.1% Triton-X, Life Technologies) for 10 min and then incubated in blocking serum (PBS-T with 5% serum from the species of the secondary antibody used) for 2 h at RT. Primary antibodies were diluted in PBS-T, applied overnight and incubated at 4 °C. Antibodies were washed 3× for 15 min at RT with PBS-T, and secondary antibodies were then applied, incubating for 2 h at RT. Cells were washed 3× with PBS-T, stained with DAPI for 15 min and finally washed with PBS 2× for 10 min. Secondary antibodies were donkey or goat Alexa Fluor 488, Alexa Fluor 568 or Alexa Fluor 647 conjugated (Life Technologies); they were all diluted 1:500 in PBS-T. The primary antibodies used are described in Table 2. Images were acquired using an Olympus IX71 inverted microscope, equipped with a DP30BW black and white digital camera or a Zeiss LSM5 Pascal Confocal Microscope situated on an air-actuated self-leveling table with Argon 458-, 488-, 514-nm and He 543-, 633-nm lasers with the capability to create topological and 3-dimensional images of cells and software for imaging/documentation systems. Z-stacks were acquired using a Zeiss Plan-Neofluar 40×/1.3 Oil objective. Fluorescent colors were digitally applied using the Olympus software DP Manager. Image quantitation was performed in ImageJ Version 2.0.0-rc-43/1.5 g. All quantitation was performed by measuring mean gray values inside Regions of Interest (ROIs) created automatically using the Nucleus counter plugin and corresponding to indicate cell markers in the channels measured. Statistics were performed in Prism 6 for Mac OS X Version 6.0 g using either ordinary one-way ANOVA with Dunnett’s multiple comparisons test or the unpaired *t*-test.

### 4.6. Histone Isolation, Western Blotting and Protein Quantification

Histones were isolated from ESC cultures using a previously-published protocol [36]. Briefly, cell cultures were extracted in lysis buffer containing 0.5% NP-40, 1.5 mM MgCl_2_, 1 mM KCl, 10 mM Tris-HCl (all from Sigma-Aldrich) supplemented with protease and phosphatase inhibitor cocktails. The lysis was performed on ice with mild vortexing, followed by centrifugation to pellet cell nuclei. Nuclei were then acid extracted with 0.4 N H_2_SO_4_ by rocking overnight. The next day, insoluble debris was pelleted and discarded, and the proteins were precipitated using 100% TCA solution for 1 h at 4 °C, followed by high-speed centrifugation to pellet the histone fraction. Pellets were washed 2× with HCl-acidified acetone and pelleted after each wash. Tubes were then allowed to air-dry, and the histone protein samples were resuspended in TBS containing protease and phosphatase inhibitor cocktail. Extracted histones were quantified using the Bradford protein assay (Bio-Rad Laboratories, Hercules, CA, USA), and 10 µg of purified histones were electrophoresed on denaturing SDS polyacrylamide gels. Gels were transferred to PVDF membranes and blocked for 1 h in 5% (*wt*/*vol*) skim milk in TBS-Tween. Following blocking, membranes were incubated with primary antibodies: anti-histone H3 AcK9 (Abcam, Cambridge, MA, USA, Catalogue Number.: ab4441), anti-histone H3 Me3K9 (Abcam, Catalogue Number.: ab8898), anti-histone H3 Me3K27 (Abcam, Catalogue Number: ab6002) or anti-histone H3 (Abcam, Catalogue Number: ab1791). Primary antibody incubations were performed overnight, followed by washing 5 × 5 min with TBS-Tween. Membranes were incubated with either anti-rabbit- or anti-mouse-HRP conjugated secondary antibodies for 1 h at RT, followed by washing 5 × 5 min in TBS-Tween. Blots were developed by incubating blots with ECL chemiluminescent reagent (Amersham, Little Chalfont, UK) and exposing to X-ray film. Protein quantification: Western blots were quantified by measuring the average pixel intensity using ImageJ. The pixel intensity of the bands was normalized to ESC expression, and bands were expressed relative to total levels of the loading control histone H3. Statistical analysis of two independent experiments was performed using one-way ANOVA with Dunnett’s test, comparing all treatment groups relative to ESC.

## Figures and Tables

**Table 1:** List of primers used for qRT-PCR experiments.

Gene	Forward Primer	Reverse Primer
*EHMT1*	GCTTCAGAAGGTGCTCCTCATG	CTGAACCAGCATGTGGCAGATG
*SUV39H1*	CCGCCTACTATGGCAACATCTC	CTTGTGGCAAAGAAAGCGATGCG
*SUV39H2*	CCATAAATGCTGGAGAAGAGCTG	GGTAACCTCTGCAAGTCACAGC
*EED*	GACGAGAACAGCAATCCAGACC	TCCTTCCAGGTGCATTTGGCGT
*MYST1*	TGGAGCCGTTCGTCTTTTAC	ATGAGGAACTTCCCGTAGCC
*MYST2*	GACAACAACAGGCATGCAAC	CCGTGTGTTCCCATAGGTCT
*MYST3*	CAAGGCTGCCCAAATTGTAT	ATCTCATTGGCAGGAGGATG
*MYST4*	GACCGCAGTACAGGGTCAAT	TCATGGGGTAGAAGGCTGAC
*MYST5*	GGCTGAGGACAGCTCAAAAA	CCGGATCCCTTCTCACTGTA
*CREBBP*	GCCACGTCCCTTAGTAACCA	CCCCAAGTGTCCCTGATCTA
*EP300*	CGCTTTGTCTACACCTGCAA	TGCTGGTTGTTGCTCTCATC
*HDAC1*	GATCTGCTCCTCTGACAAACGAA	CCCTCTCCCTCCTCTTCAGAA
*HDAC2*	TGGAGGAGGTGGCTACACAAT	AATCTCACAATCAAGGGCAACTG
*HDAC3*	TCTACCTCACTGACCGGGTCAT	ACCTGTGCCAGGGAAGAAGTAA
*HDAC8*	GGATCCCATGTGCTCCTTTA	ATAGCCTCCTCCTCCCAAAA
*HDAC11*	GAGCTGGCCCTTCCTCTACT	CTATGGGCTGGTGACTTCGT
*EHMT2*	GCCCGTTACTATGGCAACAT	GTCAAACCCTAGCTCCTCCC
*EZH1*	GTGGATGCTACTCGGAAAGG	CCCCACGTACTTGAGAGCAT
*EZH2*	TGCTATGCAAAAGTTATGATGGTT	AGTCTGGATGGCTCTCTTGG
*OCT4*	GAAGGAGAAGCTGGAGCAAA	CATCGGCCTGTGTATATCCC
*PAX6*	CCCAGCCAGACCTCCTCATA	CTTCCGGGAACTTGAACTGG
*OLIG2*	AGCGCTGTCTGGCTTTAACCT	ATGCACACAGCGGTACCTTTT
*NKX2.2*	GAACCCCTTCTACGACAGC	GGTCTCCTTGTCATTGTCCG
*MBP*	TAGGTACAGGGGCAAGTGG	TCACAAGGGATTCAAGGGAG
*GAPDH*	TGTTGCCATCAATGACCCCTT	CTCCACGACGTACTCAGCG

**Table 2:** Antibodies used in immunofluorescent stainings.

Target	Species	Company	Catalogue #	Dilution
H3-Ac	Rb	EMD Millipore	06-599	1:400
H3-K9Ac	Rb	Abcam	AB4441	1:500
H3-triMeK9	Rb	Abcam	AB8898	1:500
H3-triMeK27	Rb	EMD Millipore	07-449	1:500
PAX6	Rb	Covance	PRB-278P	1:250
NESTIN	Ms	EMD Millipore	MAB5326	1:1000
OLIG2	Ms	EMD Millipore	MABN50	1:500
SOX1	Gt	R & D Systems	AF3369	1:300
SOX10	Gt	R & D Systems	AF2864	1:100
O4	Ms	Kind gift	James Goldman	1:30
MBP	Rat	EMD Millipore	MAB386	1:200
PDGFRα	Ms	R & D Systems	MAB322	1:100
NKX2.2	Ms	DSHB	74.5A5	1:50

## Abbreviations

hESCs	human embryonic stem cells
hiPSC	human induced pluripotent stem cells
NSCs	neural stem cells
OPCs	oligodendrocyte progenitor cells
Im. OL	immature oligodendrocytes
OL	Oligodendrocytes
EZH1	Enhancer Of Zeste 1 Polycomb Repressive Complex 2 Subunit
EZH2	Enhancer Of Zeste 2 Polycomb Repressive Complex 2 Subunit
CREBBP	cAMP response element binding protein
EP300	E1A binding protein P300
EED	Embryonic Ectoderm Development
SUV39H1	Suppressor Of Variegation 3-9 Homolog 1
SUV39H2	Suppressor Of Variegation 3-9 Homolog 2
EHMT2	Euchromatic Histone-Lysine N-Methyltransferase 2
RA	retinoic acid
SAG	smoothened agonist
PDGF	platelet-derived growth factor
IGF-1	insulin-like growth factor
HGF	hepatocyte growth factor
T3	triiodothyronine
NT3	neurotrophin 3
HAT	histone acetyltransferase
HDAC	histone deacetylase
HMT	histone methyl transferase
H3ac	histone 3 acetylation
H3K9ac	histone 3 lysine 9 acetylation
H3K9me3	histone 3 lysine 9 trimethylation
H3K27me3	histone 3 lysine 27 trimethylation
CNS	central nervous system
MS	multiple sclerosis
MBP	myelin basic protein
